# Knowledge, attitudes, and practices in cardiometabolic health among adolescents and adults in HIV care in Dar es Salaam, Tanzania

**DOI:** 10.3389/fpubh.2026.1851673

**Published:** 2026-08-12

**Authors:** Peter M. Karoli, Grace Shayo, Gibson B. Kagaruki, Claudia Hawkins, Mary Mayige, Caroline Soka, Sahera Dirajlal-Fargo, Japhet Killewo, Lisa R. Hirschhorn

**Affiliations:** 1 National Institute for Medical Research-Tanzania (NIMR), Dar es Salaam, Tanzania; 2 Muhimbili University of Health and Allied Sciences (MUHAS), Dar es Salaam, Tanzania; 3 Department of Medicine, Robert J Havey Institute for Global Health, Feinberg School of Medicine, Northwestern University, Chicago, IL, United States; 4 Paediatric Infectious Disease, Ann &Robert Lurie Children‘s Hospital, Northwestern University Feinberg School of Medicine, Chicago, IL, United States; 5 Department of Medical Social Sciences, Robert J Havey Institute for Global Health, Feinberg School of Medicine, Northwestern University, Chicago, IL, United States

**Keywords:** adolescent, adult, attitudes, cardiometabolic diseases, HIV, knowledge, practice, Tanzania

## Abstract

**Background:**

Although integration of cardiometabolic conditions (CMC) prevention into HIV care is increasingly prioritized, limited evidence exists on the CMC knowledge, attitudes, and practices (KAP) of people living with HIV (PLWH). We examined cardiometabolic KAP among PLWH to inform age-appropriate interventions aimed at improving KAP and reducing CMC risk.

**Methods:**

A facility-based cross-sectional survey was conducted from April to May 2025 among adolescents (13–19 years) and adults (≥20 years) living with HIV attending six randomly selected HIV care and treatment clinics in Dar es Salaam, Tanzania. Participants were recruited consecutively. Knowledge, attitudes, and practices were assessed using a questionnaire adapted from the WHO STEPwise approach and the Global Physical Activity Questionnaire. KAP scores were categorized using modified Bloom’s cut-off criteria. Factors associated with KAP were assessed using generalized Poisson mixed-effects regression models accounting for clustering by health facility.

**Results:**

We enrolled 488 participants: 51.0% (249) were female; 20.1% (98) were adolescents (median age 17 years, IQR 14–19), and 79.9% (390) were adults (median age 41 years, IQR 32–49). Adolescents demonstrated higher knowledge of CMC risk factors than adults (75.3% vs. 55.9%, *p* < 0.001). Compared with participants with secondary education or higher, those with no formal education (aPR = 2.08, 95% CI: 1.52–2.84) and primary education (aPR = 1.60, 95% CI: 1.22–2.09) had a higher prevalence of low CMC knowledge. Negative attitudes towards CMC prevention were more prevalent among participants attending clinics in Kigamboni Municipality than Ubungo Municipality (aPR = 1.39, 95% CI: 1.25–1.54) and among divorced, separated or widowed participants than those who were married or cohabiting (aPR = 1.45, 95% CI: 1.10–1.90). Adolescents reported higher physical activity (94.9% vs. 85.1%, *p* = 0.01) but poorer dietary practices and lower uptake of blood glucose screening than adults (all *p* < 0.001). Poor CMC-related practices were more prevalent among participants attending dispensaries than hospitals (aPR = 1.97, 95% CI: 1.67–2.32) and among adolescents than adults (aPR = 1.73, 95% CI: 1.11–2.69).

**Conclusion:**

Sociodemographic factors were associated with differing KAP for CMCs risk reduction. Strengthening HIV services with age-specific strategies to improve cardiometabolic knowledge, attitudes, and preventive practices may support healthier behaviours and strengthen integrated cardiometabolic prevention within HIV care.

## 1 Introduction

The growing population of people living with HIV (PLWH) in sub-Saharan Africa faces a dual burden: managing lifelong HIV infection while increasingly at risk of early onset cardiometabolic conditions (CMCs) such as hypertension, dyslipidaemia, insulin resistance, and obesity (1, 2). These conditions are now widely recognized as comorbidities linked not only to the direct effects of HIV but also to the long-term use of antiretroviral therapy (ART) (3). As ART has substantially improved survival among people living with HIV, cardiometabolic diseases have emerged as an increasingly important cause of morbidity (4). Consequently, integrating cardiometabolic prevention into routine HIV care has become a growing priority (5, 6). Understanding patient-level knowledge, attitudes, and preventive practices (KAP) is an important component of this effort because these factors influence engagement with screening, adoption of healthy behaviours, and the implementation of patient-centred prevention strategies. Although previous studies have assessed cardiometabolic knowledge and perceptions among adults living with HIV, evidence remains limited for adolescents and direct comparisons between adolescents and adults are scarce (5, 7).

Emerging evidence indicates that cardiometabolic dysfunction begins earlier and may progress more rapidly in PLWH (8–10). This early onset of risk can compromise long-term health outcomes, leading to increased morbidity and mortality in young adulthood and as PLWH age. In Tanzania, where HIV prevalence remains high and the burden of non-communicable diseases (NCDs) continues to rise, there is growing recognition of the need to strengthen integrated service delivery models that concurrently address both infectious and chronic conditions (3, 5, 9, 11). For both adult and adolescents, the national HIV care guidelines recommend routine screening for CMCs including blood pressure measurement at each clinic visit, BMI assessment, while dyslipidaemia and blood glucose testing at least once per year, to support early detection and management (12). Despite these policy recommendations, previous studies have reported inconsistent implementation of routine cardiometabolic screening within HIV care in Tanzania, particularly in lower-level health facilities, owing to limitations in health system readiness, including shortages of equipment, provider training, and implementation capacity. These reported health system challenges may contribute to missed opportunities for early identification and management of cardiometabolic risk among people living with HIV. However, these health system factors were not directly assessed in the present study, which focused on patient-level knowledge, attitudes, and practices (13, 14).

Adolescents represent a particularly vulnerable subgroup to both the development and early progression of NCDs due to physiological and psychosocial transitions, limited engagement with health systems, and the complexities of transitioning from paediatric to adult care (5) Yet, despite their growing numbers, and opportunity to intervene early in prevention of CMCs, they remain underrepresented in research and policy discussions surrounding NCD prevention in HIV care (5, 15). Adolescents who have acquired HIV perinatally or during early childhood face a distinct and elevated risk of developing cardiometabolic conditions. In this group, long-term exposure to HIV, chronic inflammation, and ART, including older, more toxic regimens initiated during critical periods of growth and immune development, contribute to early onset CMCs (16). For example, in a Tanzanian cohort of perinatally HIV-infected adolescents in Dar es Salaam, nearly one in three had impaired fasting glucose, and hypertension was also observed (17). This combination of risk (early HIV/ART exposure, in-utero ART/HIV exposure, growth/immune-system disruption) distinguishes adolescents and young adults living with HIV from older adults and underscores the need for age-tailored screening and preventive interventions in this population (8–10).

Understanding patient-level knowledge of CMCs, attitudes toward prevention, and health behaviours, particularly related to diet and physical activity, is crucial for developing effective interventions to reduce CMC-associated risk (5, 18). While prior studies have documented these factors among adult PLWH in sub-Saharan Africa there is a notable dearth of evidence focusing on adolescents and youth, an age group that may exhibit substantially different patterns of risk perception, autonomy, and behaviour (16). This is significant as effective interventions in adolescents and young adults may be more impactful at mitigating future CMC risks.

Previous studies among people living with HIV have reported important gaps in cardiometabolic and non-communicable disease-related knowledge, risk perception, and preventive practices despite regular engagement with HIV care services. In Tanzania, Kagaruki et al. found that although awareness of hypertension and diabetes was generally high among adults receiving HIV care, detailed knowledge of disease risk factors and preventive behaviours remained limited (11). Similarly, Kamkuemah et al. reported missed opportunities for preventing non-communicable diseases among adolescents and young adults living with HIV in South Africa, highlighting important gaps in lifestyle counselling and preventive care within routine HIV services (5). However, most available studies have focused either on adults or on adolescents alone, and evidence directly comparing cardiometabolic knowledge, attitudes, and practices between adolescents and adults living with HIV remains scarce, particularly in sub-Saharan Africa (4, 5, 18, 19).

This study sought to address this gap by comparing CMC-related knowledge, attitudes, and practices among adolescents and adults living with HIV attending HIV Care and Treatment Clinics (CTCs) in urban and peri-urban settings of Dar es Salaam, Tanzania, and by identifying sociodemographic factors associated with poor cardiometabolic knowledge, attitudes, and practices. The findings are intended to identify gaps in cardiometabolic knowledge, attitudes, and practices that may inform the design and evaluation of patient-centered, integrated strategies to strengthen cardiometabolic prevention within HIV care programs, particularly for adolescents living with HIV (5).

## 2 Methods

### 2.1 Study design and setting

We conducted a cross-sectional study between April and May 2025 in CTC sampled from two municipal councils of Dar es Salaam, Tanzania: Ubungo and Kigamboni. The study targeted PLWH aged 13 years or older attending HIV care and treatment clinics. The lower age limit of 13 years was specified *a priori* in the study protocol to focus on adolescents with sufficient cognitive and psychosocial maturity to reliably complete the self-reported KAP questionnaire and respond to questions related to lifestyle behaviours and cardiometabolic health. Our primary aim was to evaluate KAP regarding cardiometabolic risk reduction, including diet and physical activity among adolescents and adults living with HIV. This cross-sectional study was reported in accordance with the Strengthening the Reporting of Observational Studies in Epidemiology (STROBE) guidelines for cross-sectional studies, as detailed in the Appendix 1 (20).

### 2.2 Sampling design, sample size calculation, and distribution

We used a stratified sampling approach to select six HIV Care and Treatment Clinics (CTCs) from 48 public CTCs (30 in Ubungo and 18 in Kigamboni Municipal Councils). Facilities were first screened for eligibility and required to have at least 100 active clients receiving HIV care and a minimum of three years of continuous HIV service delivery to ensure sufficient participant volume and stable HIV services during the study period. From the eligible facilities, two hospitals, two health centres, and two dispensaries were randomly selected to represent the different levels of HIV service delivery.

Ubungo Municipal Council is a densely populated urban area with a high concentration of health facilities and people living with HIV (PLWH), whereas Kigamboni Municipal Council is predominantly peri-urban, with fewer facilities and lower client volumes. Including facilities from both municipalities enabled the study to capture a broader range of service delivery contexts and patient characteristics.

Based on routine programme data from the six selected CTCs, 11,182 PLWH were receiving HIV care, of whom 71.9% were enrolled in Ubungo and 28.1% in Kigamboni (Appendix Table 2). The minimum sample size was calculated using the single population proportion formula, assuming a prevalence of adequate cardiometabolic knowledge of 50%, a 95% confidence level, and a 5% margin of error. A prevalence of 50% was selected because no previous study had estimated cardiometabolic knowledge among adolescents and adults living with HIV in Tanzania, providing the most conservative sample size estimate. This yielded a minimum sample of 385 participants, which was inflated using a design effect of 1.25 to account for clustering across health facilities, resulting in a required sample of 481 participants. Recruitment was conducted using consecutive sampling, and all eligible participants presenting during the study period were enrolled until data collection was completed, resulting in a final sample of 488 participants.

To ensure adequate representation of the peri-urban municipality while avoiding substantial imbalance between municipalities, the sample was allocated in a 60:40 ratio between Ubungo and Kigamboni, corresponding to target sample sizes of 289 and 192 participants, respectively (Appendix Table 2).

Because adults constitute the majority of PLWH receiving HIV care in sub-Saharan Africa, the study aimed to recruit approximately 20% adolescents (13–19 years) and 80% adults (≥20 years), consistent with the age distribution of clients attending HIV care services in the study setting and the broader regional HIV epidemiology. Accordingly, the minimum target sample comprised 96 adolescents and 385 adults.

### 2.3 Data collection

A structured questionnaire was developed to collect data on cardiometabolic knowledge, attitudes, and practices (KAP), together with clinical, socioeconomic, and demographic characteristics of people living with HIV attending six Care and Treatment Clinics (CTCs) selected from two municipalities in Dar es Salaam.

### 2.4 Tool development

The questionnaire included selected questions from the WHO STEPwise Approach to NCD Risk Factor Surveillance (WHO STEPS), the Global Physical Activity Questionnaire (GPAQ), and a previously published questionnaire developed by Kagaruki et al. to assess knowledge and perceptions of hypertension and diabetes among people living with HIV in Tanzania. These instruments had previously been translated into Kiswahili and applied in the Tanzanian context. Selected questions were used to assess the study outcomes, and minor contextual modifications were made where necessary to align the questionnaire with the objectives of the present study while preserving the original intent of the items (11, 18, 21, 22). The questionnaire was pretested before data collection to assess clarity, comprehension, and administration procedures, and minor wording modifications were made accordingly. The survey was designed to assess cardiometabolic knowledge, attitudes, and practices (KAP), together with participants’ sociodemographic characteristics and household socioeconomic status.

### 2.5 Sociodemographic and biomedical measurements

Participants’ age, sex, level of education, occupation, marital status and clinical information were recorded as part of the survey. We assessed and recorded blood glucose and blood pressure measurements for all participants, including adolescents, as these screenings are routinely recommended in the national HIV management guidelines (12). Household socio-economic status (SES) was assessed using 10 items adapted from the Tanzania Demographic and Health Surveys (21, 22). Medical records were reviewed to extract participants’ height and weight, most recent blood glucose and blood pressure measurements recorded within the previous 12 months. In cases where these clinical measurements were missing from the records, the data point was recorded as missing and excluded from any analyses requiring that variable.

### 2.6 Outcome measures

#### 2.6.1 Knowledge, attitudes, and practice

The primary outcome was participants’ KAP related to CMCs. The tool covered three domains: The knowledge domain comprised four items assessing participants’ knowledge of cardiometabolic disease risk factors, the role of diet and physical activity in disease prevention, and the recommended daily intake of fruits and vegetables according to established dietary recommendations. The knowledge of fruit and vegetable intake was assessed by asking participants to identify the recommended daily number of servings based on the WHO STEPwise Approach to NCD Risk Factor Surveillance (WHO STEPS). A serving was defined according to the WHO STEPS protocol as one standard portion of fruit or vegetables. The attitudes (11 Likert-scale items on diet, physical activity, and HIV lifestyle management). Practice was assessed using seven binary items covering dietary habits, physical activity, tobacco use, uptake of recommended cardiometabolic screening, and engagement with healthcare providers. The practice domain was designed to capture participants’ engagement in recommended cardiometabolic preventive behaviours, including both lifestyle practices and preventive health-seeking behaviours. Clinical screening practices were assessed by asking participants whether they had ever had their blood glucose measured and whether they had ever had their blood pressure measured by a doctor or other healthcare worker. Participants were also asked whether they had discussed diet and physical activity with a healthcare provider, as an indicator of engagement with preventive counselling and health education. These items were intended to capture participants’ engagement with recommended preventive healthcare services rather than the frequency or timing of these interactions. Dietary practices were assessed using standardized WHO STEPS questions that asked participants to report the number of days per week they consumed fruits and vegetables and the number of servings typically consumed on those days. A serving was defined according to the WHO STEPS protocol as one standard portion of fruit or vegetables. Table 1 summarizes the assessment domains, scoring criteria, and categorization, with specific survey items provided in Appendix Table 3. Attitude and practice scores were categorized using modified Bloom’s cut-off criteria, which are widely used in KAP studies to classify respondents according to the proportion of the maximum attainable score. Scores <60% were classified as negative attitude or poor practice, scores of 60–79% as moderate, and scores ≥80% as positive attitude or good practice. For regression analyses, the moderate and positive categories were combined and compared with the poor/negative category to generate binary outcomes (Table 1) (23–32). This classification facilitates comparability across studies by translating raw scores into meaningful performance categories and has been widely applied in KAP research to identify areas requiring targeted interventions (23–32). Because the knowledge domain comprised only four dichotomous items (score range: 0–4), modified Bloom’s cut-off criteria were not applied. Instead, participants scoring 0–1 correct responses were classified as having low knowledge, while those scoring 2–4 correct responses were classified as having high knowledge. This approach was considered more appropriate given the limited score range. In contrast, the attitude and practice domains included a larger number of items with wider score distributions and were therefore categorized using modified Bloom’s cut-off criteria.

**Table 1:** Overview of KAP assessment domains, scoring criteria, and categorization.

Domain	No. of items	Areas covered	Scoring	Categorization
Knowledge	4	1. Risk factors for cardiometabolic diseases 2. Role of diet and physical activity in risk reduction 3. Knowledge of the recommended daily fruit intake (≥5 servings/day) 4. Knowledge of the recommended daily vegetable intake (≥5 servings/day)	1 point per correct response. Total score: 0–4	Low knowledge: 0–1 High knowledge: 2–4
Attitude	11	4-point Likert scale (strongly agree, agree, disagree, strongly disagree) on diet, physical activity, and HIV lifestyle management	1 for agree/strongly agree on positive statements or disagree/strongly disagree on negative statements Total score: 0–11, Converted to percentage (score/11 × 100)	Poor: <60% Moderate: 60–79.9% Good: ≥80% Dichotomized to: Negative attitude <60% Positive attitude≥60% For multivariable analysis.
Practice	7	Binary items (yes/no) assessing: 1. Fruit intake ≥5 days/week 2. Vegetable intake ≥5 days/week 3. Physical Activity ≥30 min on ≥3 days/week 4. Never smoked tobacco 5. Ever had blood glucose measured by a healthcare provider 6. Ever had blood pressure measured by a healthcare provider 7. Discussed diet/Physical activity with healthcare workers	1 point for each positive practice Total score: 0–7, Converted to percentage (score/7 × 100)	Poor: <60% Moderate: 60–79.9% Good: ≥80% Dichotomized to: Poor <60% Moderate/Good ≥60%

### 2.7 Data collection procedures

Trained research assistants collected data using tablets loaded with Open Data Kit (ODK) software, an open-source mobile data collection platform widely used in global health research (33–35). Consecutive sampling was employed to recruit participants as they attended routine HIV services at the study clinics, where exit interviews were subsequently conducted. Questionnaires were administered in Swahili to ensure comprehension. To maintain confidentiality and privacy, interviews were conducted in a private setting, and all collected data were deidentified and securely stored in a password protected database.

### 2.8 Data management

Data quality was prioritized throughout the study to ensure that findings were reliable and ethically sound. To maintain data integrity, consistency checks were built into the Kobo Toolbox system to prevent missing or implausible entries. The system included controls for variable ranges, skip patterns, duplicate entries, and intra- and inter-module consistency, minimizing errors at the data entry stage. Additionally, the Data Manager ensured that daily data collection reports aligned with the data uploaded to the central server.

After data collection, the dataset was reviewed for completeness, consistency, outliers, and missing values. Logical checks were performed to ensure internal consistency, and implausible values were verified against the original records where possible. Missing data were assessed for each study variable. Participants with missing values for a specific variable were excluded only from analyses involving that variable (complete-case analysis), while available observations were retained for all other analyses. No imputation of missing values was undertaken. Before downloading, field tablets were reviewed to confirm all questionnaires had uploaded successfully, with any failures resolved through re-synchronization. The final dataset was then downloaded in CSV format and imported into Stata version 17 (StataCorp, TX, United States) for analysis.

### 2.9 Data analysis

Knowledge: Each correct response was assigned a score of one, yielding a total score ranging from 0 to 4. This variable was dichotomized to low knowledge for score of 0–1 and high knowledge for those who scored 2–4 (Table 1). For attitude, the total score (0–11) was converted into a percentage and using Blooms cutoff, were classified as poor (<60%), moderate (60–79.9%), or good (≥80%) attitude. Attitudes were then dichotomized for use in the MVA as negative (<60%) or positive (≥60%). For practices, each positive practice was scored as one, summed, and converted to a percentage, with thresholds of poor (<60%), moderate (60–79.9%), or good (≥80%) practice (Table 1). Similar to attitude, moderate and good practices were combined and compared with poor practices.

Descriptive statistics were used to summarize the data including medians with interquartile ranges (IQR) for KAP score, and proportions (%), as appropriate. Household socioeconomic status (SES) was estimated using principal component analysis (PCA) based on ten household asset ownership and housing characteristic variables adapted from the Tanzania Demographic and Health Survey (DHS). These included household access to electricity, source of drinking water, type of sanitation facility, ownership of a television, radio, refrigerator, mobile phone, bicycle, motorcycle or motor vehicle, and housing construction materials (floor, roof, and wall). The first principal component was used to derive the household wealth index, which was subsequently categorized into wealth quintiles in accordance with established DHS methodology (22, 36). The first principal component was used to derive a household wealth index, which was subsequently categorized into wealth quintiles. Differences in median knowledge, attitude and practice scores between age groups were assessed using the non-parametric Wilcoxon rank-sum test. Comparative analyses of categorical variables were performed using Pearson’s chi-square test or Fisher’s exact test, as appropriate. Fisher’s exact test was applied when expected cell counts were small or when the assumptions of the chi-square test were not satisfied.

Multivariable analysis was performed using a generalized Poisson mixed-effects regression model with robust variance estimation to estimate adjusted prevalence ratios (aPRs) and corresponding 95% confidence intervals (95% CIs). Random intercepts for health facilities were included to account for clustering of participants within the six HIV Care and Treatment Clinics (CTCs). This modelling approach was selected because the prevalence of the study outcomes (low knowledge, negative attitudes, and poor practices related to diet, physical activity, and HIV-related lifestyle management) exceeded 10%, making prevalence ratios more appropriate and clinically interpretable than odds ratios from conventional logistic regression models. Prior to fitting the multivariable models, multicollinearity among explanatory variables was assessed using Variance Inflation Factors (VIFs). No evidence of problematic multicollinearity was identified, and all variables were retained in the final models. Explanatory variables with a *p*-value <0.20 in the univariable analysis, together with variables considered clinically and epidemiologically relevant based on existing evidence, were included in the multivariable models. Complete-case analysis was used for all regression models. There were no missing data for the primary KAP outcomes or covariates included in the multivariable analyses; therefore, all eligible participants were retained in the regression models. Diabetes and hypertension status were reported descriptively only because these variables had substantial missing data resulting from incomplete routine clinical screening. Statistical significance was defined as a two-sided *p*-value <0.05. Both unadjusted and adjusted prevalence ratios are presented to facilitate interpretation of the observed associations (36).

### 2.10 Human subjects and ethics approval

Ethical approval was granted by the Research Ethics Committees from Muhimbili University of Health and Allied Sciences (MUHAS-REC-02-2025-2650) and from the National Institute for Medical research Tanzania (NIMR/HQ/R.8a/Vol.IX/5188). The study was conducted in compliance with institutional guidelines and the ethical principles accordingly. Prior to participation, a written informed consent was obtained from adults, and assent along with parental or guardian consent was secured for adolescents below 18 years.

## 3 Results

As shown in Table 2, the study population (*N* = 488) included 20% adolescents (median age, 17 years; IQR, 14–19) and 80% adults (median age, 41 years; IQR, 32–49). Among adolescents, sex distribution was nearly equal (51% female and 49% male), whereas adults were predominantly female (73.3%). Comorbidities were less common in adolescents than in adults, with no reported cases of diabetes (0% vs. 9.1%, *p* < 0.001) and lower prevalence of hypertension (5.7% vs. 21.2%, *p* < 0.001), respectively.

**Table 2:** Sociodemographic and clinical characteristics of adolescents and adults living with HIV enrolled in the study (*N* = 488).

Variable	Category	Overall % (*N*)	Adolescents (13–19 years) % (*N*)	Adults (20 + years) % (*N*)
District	Ubungo	59.8 (292)	60.2 (59)	59.7 (233)
	Kigamboni	40.2 (196)	39.8 (39)	40.3 (157)
Facility level	Dispensary	13.5 (66)	14.3 (14)	13.3 (52)
	Health center	46.3 (226)	46.9 (46)	46.2 (180)
	Hospital	40.2 (196)	38.8 (38)	40.5 (158)
Age	Median (IQR)	37.0 (25–46.5)	17.0(14–19)	41.0(32–49)
Sex	Male	31.1 (152)	49.0 (48)	26.7 (104)
	Female	68.9 (336)	51.0(50)	73.3 (286)
Marital status	Single	49.7 (228)	99.0 (97)	33.6 (131)
	Married	29.1 (142)	0.0 (0)	36.4 (142)
	Divorced	6.6 (32)	0.0 (0)	8.2 (32)
	Separated	9.4 (46)	0.0 (0)	11.8 (46)
	Widowed	8.2 (40)	1.0 (1)	10.0 (39)
Education (highest completed)	No formal education	8.8 (43)	1.0 (1)	10.8 (42)
	Primary school	53.9 (263)	42.9 (42)	56.7 (221)
	Secondary school	30.9 (151)	51.0(50)	25.9 (101)
	College/university	6.4 (31)	5.1 (5)	6.7 (26)
Occupation	Employed	12.3 (60)	11.2 (11)	12.6 (49)
	Self-employed	59.4 (290)	8.2 (8)	72.3 (282)
	Unemployed*	28.3 (138)	80.6 (79)	15.1 (59)
Household Wealth index	Lowest	20.7 (101)	13.3 (13)	22.6 (88)
	Second	20.7 (101)	17.4 (17)	21.5 (84)
	Middle	21.7 (106)	26.5 (26)	20.5 (80)
	Fourth	33.4 (163)	28.6 (28)	34.6 (135)
	Highest	3.5 (17)	14.3 (14)	0.8 (3)
Self-reported hypertension % (n/N)		18.8% (85/452)	5.7% (4/70)	21.2% (81/382)
Self-reported diabetes % (n/N)		8.0 (17/213)	0.0 (0/26)	9.1% (17/187)

*Include students (1) Missing data were minimal for the primary KAP variables and covariates included in the regression analyses. However, diabetes status was unavailable for a substantial proportion of participants because blood glucose testing was not routinely performed for all participants attending the HIV Care and Treatment Clinics during the study period. Consequently, diabetes status was included only as a descriptive baseline characteristic and was not incorporated into the multivariable regression analyses. (2) The observed difference in household wealth quintiles between adolescents and adults should be interpreted in the context of the household-based wealth index. For adolescents, the wealth index primarily reflects the socioeconomic status of the parental or guardian household, whereas for adults it reflects their own household circumstances.

Just over half (59.7%) of respondents knew the risk factors for CMCs, with higher knowledge among adolescents (75.3%) than adults (55.9%, *p* < 0.01) (Table 3). Knowledge that diet and physical activity reduce CMC risk was high overall (93.2%). However, only 2.3% knew the recommended daily fruit intake (≥5 servings) and 2.1% knew the recommendation for vegetables (≥5 servings).

**Table 3:** Proportion of respondents with correct knowledge, positive attitude and good practices towards diet, physical activity, and HIV-related lifestyle management by study group.

Domain		Overall, % (*N*)	Age group		
	Expected response		Adolescents, % (*N*)	Adults, % (*N*)	*p*-value
Knowledge					
Knows risk factors for cardiometabolic diseases	Correct	59.7 (279)	75.3 (70)	55.9 (209)	<0.001
Knows diet/physical activity reduce risk of cardiometabolic diseases	Correct	93.2 (455)	91.8 (90)	93.6 (365)	0.537
Knows that a person should consume ≥ 5 servings of fruits daily	Correct	2.3 (11)	2.0 (2)	2.3 (9)	0.874
Knows that a person should consume ≥ 5 servings of vegetables daily	Correct	2.1 (10)	4.1 (4)	1.5 (6)	0.112
Overall knowledge classification					
High knowledge		55.1 (269)	68.4 (67)	51.8 (202)	0.003
Low knowledge		44.9 (219)	31.6 (31)	48.2 (188)	
Knowledge score, Median (IQR)		2 (1–2)	2 (1–2)	2 (1–2)	0.0045
Attitude					
Obesity increases the risk of cardiometabolic diseases.	Agree	12.7 (62)	26.5 (26)	9.2 (36)	<0.001
It is important to eat a healthy diet to reduce the risk of heart disease.	Agree	8.4 (41)	14.3 (14)	6.9 (27)	0.019
I believe physical activity is too difficult to maintain regularly.	Disagree	44.5 (217)	28.6 (28)	48.5 (189)	<0.001
Healthy eating is expensive and not practical for most people.	Disagree	69.1 (337)	55.1 (54)	72.6 (283)	<0.001
If someone has a family history of heart disease, they can reduce their risk by eating a healthy diet.	Agree	35.5 (173)	35.7 (35)	35.4 (138)	0.951
Regular physical activity is essential for managing my NCDs condition	Agree	7.8 (38)	9.2 (9)	7.4 (29)	0.564
I feel I have little control over my health risks	Disagree	52.3 (255)	48.0 (47)	53.3 (208)	0.341
A person should maintain excess body weight to avoid being perceived as HIV positive	Disagree	19.7 (96)	16.3 (16)	20.5 (80)	0.351
Becoming thin or slender may lead others to assume a person is HIV positive	Disagree	25.8 (126)	24.5 (24)	26.2 (102)	0.736
A glass of non-sugar juice is better than chewing the actual fruits	Disagree	26.0 (127)	20.4 (20)	27.4 (107)	0.156
It is very difficult to yourself to avoid overfeeding if delicious foods are plenty available	Disagree	38.5 (188)	45.9 (45)	36.7 (143)	0.092
Overall attitude classification					
Negative attitude		27.1 (132)	20.4 (20)	28.7 (112)	0.236
Moderate attitude		37.9 (185)	42.9 (42)	36.7 (143)	
Positive attitude		35.0 (171)	36.7 (36)	34.6 (135)	
Attitude score, Median (IQR)		8 (6–9)	8 (7–9)	8 (6–9)	0.219
Practices					
Consumes fruits ≥ 5 days/per week	Yes	38.1 (186)	22.5 (22)	42.1 (164)	<0.001
Consumes vegetables ≥ 5 days/per week	Yes	62.5 (305)	48.0 (47)	66.2 (258)	<0.001
Engages in physical activity at least 30 min, ≥ 3 days per week	Yes	87.1 (425)	94.9 (93)	85.1 (332)	0.01
Ever smoked tobacco	No	8.0 (39)	1.0 (1)	9.7 (38)	0.004
Ever had blood glucose measured by a healthcare provider	Yes	45.6 (213)	13.3 (13)	51.3 (200)	<0.001
Ever had blood pressure measured by a healthcare provider	Yes	92.6 (452)	71.4 (70)	98.0 (382)	<0.001
Ever discussed healthy diet and physical activity with a healthcare provider	Yes	38.1 (186)	22.5 (22)	42.1 (164)	<0.001
Overall practice classification					
Poor practice		62.1 (303)	83.7 (82)	56.7 (221)	<0.001
Moderate practice		24.2 (118)	12.2 (12)	27.2 (106)	
Good practice		13.7 (67)	4.1 (4)	16.1 (63)	
Practice score, median (IQR)		5 (4–6)	4 (3–5)	5 (4–6)	<0.001

Adolescents reported more incorrect attitudes than adults, including disagreeing that obesity increases the risk of cardiometabolic diseases (26.5% versus 9.2%, *p* < 0.001) and disagreeing that a healthy diet reduces heart disease risk (14.3% versus 6.9%, *p* = 0.019). Overall, 44.5% of participants agreed that physical activity was too difficult to maintain. This perception was less common among adolescents than adults (28.6% versus. 48.5%, *p* < 0.001).

For practices, overall, 38.1% of participants consumed fruits on ≥5 days per week, with lower proportion among adolescents (22.5%) compared to adults (42.1%), *p* < 0.001. Vegetable consumption on ≥5 days per week was reported by 62.5%, again lower among adolescents (48.0%) than adults (66.2%, *p* = 0.01). Physical activity for ≥30 min at least 3 days per week was reported by 87.1% and was more common among adolescents (94.9%) than adults (85.1%, *p* = 0.01). Overall, 45.6% of participants reported ever having had their blood glucose measured by a doctor or other healthcare provider. This proportion was substantially lower among adolescents (13.3%) than adults (51.3%; *p* < 0.001).

As shown in Table 4, after adjustment for clustering and other covariates, low knowledge remained independently associated with having no formal education (aPR 2.08, 95% CI 1.52–2.84), primary education (aPR 1.60, 95% CI 1.22–2.09), belonging to the lowest wealth quintile (aPR 1.53, 95% CI 1.14–2.06), and the second wealth quintile (aPR 1.48, 95% CI 1.08–2.01).

**Table 4:** Modified Poisson regression analysis of factors associated with lower knowledge, negative attitude, and poor dietary and physical activity practices.

Variable	Low knowledge			Negative attitude			Poor practice		
	uPR (95% CI)	*p*-value	aPR (95% CI)	uPR (95% CI)	*p*-value	aPR (95% CI)	uPR (95% CI)	*p*-value	aPR (95% CI)
Municipal									
Ubungo	Ref		Ref	Ref		Ref	Ref		Ref
Kigamboni	1.30 (1.21–1.40)	<0.001	1.20 (0.97–1.50)	1.54 (1.46–1.62)	<0.001	1.39 (1.25–1.54)	2.25 (1.60–3.18)	<0.001	1.58 (1.33–1.88)
Health facility type									
Hospital	Ref		Ref	Ref			Ref		Ref
Dispensary	1.28 (1.14–1.45)	<0.001	1.06 (0.79–1.42)	1.20 (0.83–1.74)	0.331		2.76 (1.98–3.83)	<0.001	1.97 (1.67–2.32)
Health centre	1.10 (0.88–1.39)	0.401	0.92 (0.73–1.16)	1.22 (0.87–1.71)	0.256		2.27 (1.46–3.52)	<0.001	1.70 (1.41–2.06)
Sex									
Male	Ref		Ref	Ref			Ref		
Female	0.90 (0.81–1.01)	0.074	0.83 (0.68–1.01)	0.94 (0.65–1.35)	0.723		0.76 (0.49–1.19)	0.235	
Age group									
≥20 years	REF		REF	REF		REF	REF		REF
13–19 years	0.66 (0.56–0.78)	<0.001	0.73 (0.50–1.06)	0.71 (0.46–1.10)	0.127	0.84 (0.46–1.54)	1.69 (1.08–2.66)	0.023	1.73 (1.11–2.69)
Marital status									
Single/never married	Ref		Ref	Ref		Ref	Ref		Ref
Married	1.15 (0.95–1.39)	0.159	0.88 (0.70–1.12)	1.25 (0.86–1.81)	0.253	1.09 (0.70–1.71)	0.70 (0.52–0.93)	0.015	0.83 (0.57–1.23)
Others	1.14 (1.00–1.30)	0.053	0.84 (0.66–1.07)	1.77 (1.59–1.98)	<0.001	1.45 (1.10–1.90)	0.71 (0.43–1.16)	0.175	0.86 (0.60–1.23)
Education level									
Secondary education	Ref		Ref	Ref		Ref	Ref		
No formal education	2.44 (1.88–3.17)	<0.001	2.08 (1.52–2.84)	2.63 (1.77–3.92)	<0.001	1.97 (1.35–2.87)	0.86 (0.46–1.63)	0.651	
Primary school	1.66 (1.30–2.12)	<0.001	1.60 (1.22–2.09)	1.99 (1.56–2.53)	<0.001	1.68 (1.31–2.15)	1.01 (0.75–1.35)	0.962	
College/University	0.85 (0.45–1.61)	0.613	0.84 (0.45–1.59)	1.42 (0.84–2.41)	0.193	1.55 (0.94–2.54)	0.64 (0.34–1.19)	0.160	
Occupation									
Employed	Ref		Ref	Ref		Ref	Ref		Ref
Self-employed	0.98 (0.87–1.10)	0.703	0.84 (0.63–1.12)	1.36 (0.98–1.89)	0.064	1.10 (0.78–1.54)	0.86 (0.72–1.03)	0.101	1.04 (0.87–1.24)
Unemployed	0.79 (0.63–1.00)	0.051	0.80 (0.56–1.14)	1.49 (1.02–2.16)	0.039	1.47 (0.84–2.56)	1.21 (1.01–1.45)	0.034	0.98 (0.81–1.17)
Wealth index									
Middle	Ref		Ref	Ref		Ref	Ref		Ref
Lowest	1.77 (1.22–2.55)	0.002	1.53 (1.14–2.06)	1.68 (1.19–2.36)	0.003	1.38 (0.98–1.94)	1.62 (1.25–2.10)	<0.001	1.40 (1.01–1.94)
Second	1.55 (1.16–2.07)	0.003	1.48 (1.08–2.01)	1.39 (1.03–1.87)	0.033	1.28 (0.96–1.71)	1.56 (1.06–2.28)	0.023	1.31 (0.91–1.90)
Fourth and highest	0.95 (0.79–1.13)	0.540	1.05 (0.76–1.46)	0.80 (0.60–1.07)	0.137	0.93 (0.63–1.37)	1.04 (0.61–1.77)	0.899	1.17 (0.81–1.69)
Knowledge level									
High knowledge							Ref		Ref
Low knowledge							1.41 (1.23–1.61)	<0.001	1.32 (1.24–1.40)
Attitude									
Positive attitude							REF		REF
Negative attitude							1.27 (0.97–1.66)	0.086	1.12 (0.86–1.46)

Negative attitudes were independently associated with residence in Kigamboni Municipality (aPR 1.39, 95% CI 1.25–1.54), being previously married (aPR 1.45, 95% CI 1.10–1.90), having no formal education (aPR 1.97, 95% CI 1.35–2.87), and primary education (aPR 1.68, 95% CI 1.31–2.15).

Poor practice was independently associated with residence in Kigamboni Municipality (aPR 1.58, 95% CI 1.33–1.88), receiving care at dispensaries (aPR 1.97, 95% CI 1.67–2.32) or health centres (aPR 1.70, 95% CI 1.41–2.06), being an adolescent (aPR 1.73, 95% CI 1.11–2.69), belonging to the lowest wealth quintile (aPR 1.40, 95% CI 1.01–1.94), and having low cardiometabolic knowledge (aPR 1.32, 95% CI 1.24–1.40).

## 4 Discussion

This study provides a comprehensive assessment of knowledge, attitudes, and practices (KAP) related to cardiometabolic conditions (CMCs) among people living with HIV (PLWH) in Tanzania, highlighting important age-, socioeconomic-, and health system-related disparities in CMC prevention. Although overall knowledge of CMCs was relatively high, important gaps remained in preventive practices, particularly healthy dietary behaviours and uptake of guideline-recommended cardiometabolic screening within HIV care services (6). Adolescents demonstrated higher knowledge of CMC risk factors and greater engagement in physical activity than adults but reported poorer dietary practices and substantially lower uptake of blood glucose screening despite national HIV guidelines recommending routine cardiometabolic risk assessment for both adolescents and adults receiving HIV care (6). After accounting for clustering by health facility, low knowledge remained independently associated with lower educational attainment and socioeconomic disadvantage, whereas negative attitudes were more prevalent among participants attending clinics in the peri-urban municipality of Kigamboni, those with lower educational attainment, and divorced, separated, or widowed individuals. Poor preventive practices were independently associated with adolescence, residence in Kigamboni, receiving care at lower-level health facilities, low cardiometabolic knowledge, and socioeconomic disadvantage. Together, these findings underscore the need for integrated, context-sensitive approaches that strengthen cardiometabolic health promotion, preventive screening, and behavioural interventions within routine HIV care, particularly for adolescents and socioeconomically disadvantaged populations.

Adolescents demonstrated higher knowledge of CMC risk factors than adults, with nearly three-quarters correctly identifying key risk factors compared with just over half of adults. This finding is consistent with school-based studies from Tanzania and other low- and middle-income countries (LMICs), where adolescents often report greater exposure to health information through school curricula, media, and health promotion campaigns (15, 37, 38). Similar observations have been reported among young people living with HIV in South Africa, where adolescents exhibited greater awareness of non-communicable disease (NCD) risk factors than older adults (5). However, after adjustment for educational attainment, socioeconomic status, and other covariates, age was no longer independently associated with knowledge, suggesting that the observed age differences were largely explained by underlying socioeconomic and educational factors rather than age alone. Importantly, higher knowledge among adolescents did not translate into healthier preventive practices, reinforcing evidence from sub-Saharan Africa that knowledge alone is often insufficient to overcome barriers such as food insecurity, limited autonomy in household food choices, and restricted access to preventive health services (4, 5, 11, 39). These findings highlight that improving cardiometabolic health among PLWH requires interventions that extend beyond health education to address the social and structural determinants of healthy behaviours.

Lower educational attainment emerged as one of the strongest independent predictors of poor cardiometabolic knowledge. Compared with participants who had secondary education or higher, those with no formal education or only primary education were significantly more likely to have low CMC knowledge, consistent with studies among PLWH in Tanzania and elsewhere showing that limited education constrains understanding of hypertension, diabetes, and other cardiometabolic risk factors (11, 18, 40). Similarly, participants in the poorest wealth quintile were more likely to have low knowledge than those in the middle wealth quintile, highlighting the influence of socioeconomic disadvantage on health literacy and access to health information (41, 42). Comparable associations between lower socioeconomic status and reduced health knowledge have also been reported in studies conducted in other resource-limited settings in sub-Saharan Africa (41, 42). Although household wealth indices may not be directly comparable between adolescents and adults because adolescents’ socioeconomic status primarily reflects that of their parents or guardians, the observed association between poverty and lower cardiometabolic knowledge remained consistent across the study population. These findings indicate that current HIV service delivery models may not adequately address the needs of socially disadvantaged populations and underscore the importance of tailoring health education strategies to individuals with lower educational attainment and socioeconomic resources.

Negative attitudes toward CMC prevention were independently associated with lower educational attainment, being divorced, separated or widowed, and receiving HIV care in Kigamboni Municipality. Participants with limited education are likely to have lower health literacy, reducing their ability to interpret risk information and translate preventive recommendations into favourable attitudes toward cardiometabolic disease prevention, consistent with previous studies from Tanzania (11, 18). Similarly, individuals who were divorced, separated or widowed may experience reduced social support, financial hardship, and psychosocial stress, all of which can adversely influence engagement with healthy lifestyles and chronic disease prevention (43–45).

The observed geographic differences in attitudes further suggest that health system and environmental contexts influence cardiometabolic prevention. Participants attending HIV clinics in Kigamboni Municipality, a predominantly peri-urban setting, had a higher prevalence of negative attitudes than those attending clinics in the more urbanized Ubungo Municipality. Similar geographic differences in cardiometabolic knowledge, attitudes, and preventive behaviours have been reported in studies conducted across urban, peri-urban, and rural settings in sub-Saharan Africa, although the direction and magnitude of these differences vary depending on local socioeconomic conditions, healthcare access, and health system capacity (46, 47). Our findings suggest that these contextual factors may exert a greater influence on preventive attitudes than urbanicity alone. Differences in access to preventive health services, healthy food environments, recreational facilities, and community-based health promotion may partly explain the observed disparities. These findings reinforce the importance of context-specific interventions that strengthen primary healthcare services, improve access to healthy lifestyle opportunities, and address structural barriers to cardiometabolic prevention within routine HIV care, particularly in underserved peri-urban communities (19, 48–50).

Despite demonstrating higher knowledge of CMC risk factors than adults, adolescents reported poorer preventive practices, particularly with respect to healthy dietary behaviours and uptake of blood glucose screening. Fewer than one-quarter reported regular fruit consumption, and only 13.3% had ever undergone blood glucose screening. Similar knowledge–practice gaps have been documented among adolescents in Tanzania and other LMICs, suggesting that awareness alone is insufficient to promote sustained healthy behaviours when structural barriers persist (37, 39). Importantly, our multivariable analysis also showed that participants with low cardiometabolic knowledge had a higher prevalence of poor preventive practices, reinforcing the critical role of health literacy in translating knowledge into action. Together, these findings suggest that improving preventive behaviours among PLWH requires interventions that combine health education with strategies addressing affordability of healthy foods, household decision-making, and access to preventive screening services.

Beyond individual-level characteristics, poor preventive practices were independently associated with receiving HIV care at health centres and dispensaries compared with hospitals. These findings suggest that differences in health system capacity and service readiness may influence the delivery and uptake of integrated cardiometabolic prevention within routine HIV care. Similar disparities in the quality of NCD prevention and management across different levels of healthcare have been reported in Tanzania and other sub-Saharan African countries, where lower-level facilities often face shortages of trained healthcare providers, limited availability of diagnostic equipment, inconsistent implementation of routine cardiometabolic screening, and fewer opportunities for comprehensive lifestyle counselling (13, 48). Although Tanzania’s national HIV guidelines recommend routine cardiometabolic risk assessment for people living with HIV, implementation may vary across levels of healthcare depending on available resources and service capacity (6). Our findings reinforce the need to strengthen the capacity of primary healthcare facilities through workforce training, reliable screening equipment, standardized risk assessment, and integrated health education to improve the quality and equity of cardiometabolic prevention within HIV care services (19, 48).

Collectively, these findings indicate that improving cardiometabolic prevention among people living with HIV requires coordinated interventions operating at individual, health system, and community levels. Individual-level strategies should strengthen cardiometabolic health literacy, particularly among people with lower educational attainment and those from socioeconomically disadvantaged households, while ensuring that health education translates into sustained preventive behaviours. For adolescents, HIV services should incorporate youth-friendly cardiometabolic risk assessment, nutrition education, family engagement, and behaviour-change support to address the observed knowledge–practice gap. At the health system level, strengthening the capacity of primary healthcare facilities, particularly health centres and dispensaries, to deliver integrated cardiometabolic screening, risk communication, and preventive counselling may improve the quality and equity of HIV care. Finally, multisectoral collaboration involving the health, education, agriculture, and local government sectors is needed to improve access to healthy foods, supportive environments for physical activity, and equitable delivery of cardiometabolic prevention services within routine HIV care (19, 48, 50, 51).

### 4.1 Strengths and limitations

This study has several strengths. It used standardized, previously validated WHO STEPwise Approach to NCD Risk Factor Surveillance (WHO STEPS) and Global Physical Activity Questionnaire (GPAQ) instruments, facilitating comparison with national and international studies. Inclusion of both adolescents and adults receiving HIV care enabled age-specific comparisons and provided insights into cardiometabolic knowledge, attitudes, and preventive practices across different stages of the life course. Furthermore, the use of generalized Poisson mixed-effects regression models with random intercepts for health facilities accounted for clustering of participants within study sites, thereby strengthening the validity of the estimated associations.

However, several limitations should be considered when interpreting these findings. First, the cross-sectional design precludes causal inference, and the study was powered to estimate the overall prevalence of cardiometabolic knowledge rather than detect differences across all subgroup analyses. Consequently, some subgroup comparisons, particularly among smaller categories, may have been underpowered. Second, participants were recruited consecutively from HIV Care and Treatment Clinics (CTCs) with at least 100 active clients and three years of continuous service delivery, limiting generalizability to smaller or newer facilities, individuals not engaged in HIV care, and adolescents younger than 13 years. Although the planned age-stratified sample was achieved, the smaller adolescent sample may have reduced the precision of age-specific estimates.

Third, several practice measures, including previous blood pressure and blood glucose screening, were self-reported and not verified against medical records, making them susceptible to recall bias. Uptake of cardiometabolic screening may also reflect health system factors, such as service availability and provider practices, rather than individual health-seeking behaviour alone. In addition, household food security and school enrolment among adolescents were not assessed.

Fourth, although the questionnaire was adapted from previously validated instruments used in Tanzania, the knowledge and practice domains covered only selected aspects of cardiometabolic health, and the modified Bloom’s cut-off may have oversimplified participants’ knowledge levels. We also did not reassess the psychometric properties of the attitude scale in the current study population.

Finally, data on viral suppression, ART regimen, and duration on ART were unavailable. In addition, diabetes status was unavailable for a substantial proportion of participants because blood glucose testing was not routinely performed during the study period. Consequently, diabetes was reported descriptively only and excluded from multivariable analyses, and its prevalence may have been underestimated.

## 5 Conclusion

This study identified important disparities in cardiometabolic knowledge, attitudes, and preventive practices among people living with HIV in Tanzania. Although adolescents demonstrated higher knowledge of cardiometabolic risk factors and greater engagement in physical activity, they reported poorer dietary practices and lower uptake of blood glucose screening than adults. Lower educational attainment, socioeconomic disadvantage, residence in a peri-urban municipality, and receiving care at lower-level health facilities were independently associated with poorer cardiometabolic knowledge, attitudes, and preventive practices. These findings underscore the need to strengthen integrated cardiometabolic prevention within HIV care through age-responsive, context-specific, and equity-oriented strategies that enhance health literacy, expand preventive screening, and strengthen the capacity of primary healthcare facilities to deliver comprehensive cardiometabolic services.

## Data Availability

The dataset supporting the findings of this study is available upon reasonable request and completion of a Data Transfer Agreement (DTA) with the National Institute for Medical Research (NIMR) Ethics Board, in accordance with Tanzanian regulatory requirements. Interested researchers may contact the corresponding author, who will facilitate the DTA process as required by national data governance policies. Data will be shared following approval from NIMR to ensure compliance with ethical and legal standards for the protection of participant information.
