# Supplementary material for: Knowledge, attitudes, and practices in cardiometabolic health among adolescents and adults in HIV care in Dar es Salaam, Tanzania
Source: Front Public Health. 2026 Aug 12;14:1851673. doi: 10.3389/fpubh.2026.1851673 (PMC13506751; doi:10.3389/fpubh.2026.1851673)
Supplement: Supplementary file 2 [file Supplementary_file_2.docx]

**APPENDIX:**

1. ***Table 2*: Sample size distribution using proportional allocation technique.**

| Municipal | Level | Number of clients | Sample size | 13-19Yrs | 20+ Yrs |
| --- | --- | --- | --- | --- | --- |
| Ubungo | Health Centre | 2,491 | 90 | 18 | 72 |
|  | District Hospital | 4,880 | 176 | 35 | 141 |
|  | Dispensary | 665 | 24 | 5 | 19 |
|  | **Total** | **8,036** | **289** | **58** | **231** |
| Kigamboni | District Hospital | 280 | 17 | 3 | 14 |
|  | Health Centre | 2,200 | 134 | 27 | 107 |
|  | Dispensary | 666 | 41 | 8 | 33 |
|  | **Total** | **3,146** | **192** | **38** | **154** |
