# Supplementary material for: Knowledge, attitudes, and practices in cardiometabolic health among adolescents and adults in HIV care in Dar es Salaam, Tanzania
Source: Front Public Health. 2026 Aug 12;14:1851673. doi: 10.3389/fpubh.2026.1851673 (PMC13506751; doi:10.3389/fpubh.2026.1851673)
Supplement: Supplementary file 3 [file Supplementary_file_3.docx]

**APPENDIX:**

1. ***Table 3*: Domain and interview questions**

| Domain | Questions | Assessment method |
| --- | --- | --- |
| **Knowledge** | 1. **(a) Do you know some risk factors for cardiometabolic diseases?**    a. Yes    b. No    c. 13**(b)If yes, List as many as you can.** 2. **Can diet and physical activity reduce the risk of cardiometabolic diseases?**    a. Yes    b. No    c. Don’t know 3. **How many servings of fruits should a person consume daily to reduce NCS disease risk?**    a. ____________ servings of fruit 4. **How many servings of vegetables should a person consume daily to reduce NCS disease risk?**    a. ____________ servings of vegetables | **Binary items (correct/incorrect) assessing:**  1.Risk factors for cardiometabolic diseases  2.Role of diet/PA in risk reduction  3.Daily fruit intake (≥5 servings)  4.Daily vegetable intake (≥5 servings) |
| **Attitude** | (Use a 4-point Likert scale: 1 = Strongly agree, 2 = Agree, 3 = Disagree, 4 = Strongly disagree)   1. **Obesity increases the risk of cardiometabolic diseases** 2. **It is important to eat a healthy diet to reduce the risk of heart disease.** 3. **I believe physical activity is too difficult to maintain regularly.** 4. **Healthy eating is expensive and not practical for most people.** 5. **If someone has a family history of heart disease, they can reduce their risk by eating a healthy diet.** 6. **Regular physical activity is essential for managing my HIV condition.** 7. **I feel I have little control over my health risks.** 8. **If somebody is fat must maintain his/her body structure otherwise can be HIV positive.** 9. **To become thin and slender is not good because people will consider one to be HIV positive.** 10. **A glass of non-sugar juice is better than chewing the actual fruits** 11. **It is very difficult to yourself to avoid overfeeding if delicious foods are plenty available** | **- Binary categories:**  Positive attitude: "1" if "agree/strongly agree" on positive statements  or  "disagree/strongly disagree" on negative statements  - Negative attitude: "0" otherwise |
| **Behaviour (Practice)** | 1. **In a typical week, how many days do you eat fruit?**   ______ days   2. **In a typical week, how many days do you eat vegetables?**   ______ days   3. **Do you engage in any form of physical activity (walking, exercise, chores)?**    a. Yes    b. No   **If yes, how many days per week do you engage in physical activity for at least 30 minutes?** ______ days  **How often do you engage in vigorous physical activity (e.g., running, cycling)?** ______ days per week   4. **Do you use any tobacco products to smoke, or chew?**   **1. Yes**  **2. No**  **If yes, do you currently use tobacco products daily?**  **1. Yes**  **2. No**   5. **Have you ever had your blood sugar measured by a doctor or other health worker?**   **1. Yes**  **2. No**   6. **Have you ever had your blood pressure measured by a doctor or other health worker?**   **1. Yes**  **2. No**   7. **Have you ever discussed diet and physical activity with your healthcare provider?**    1. **Yes**    2. **No** | **Binary items assessing:**  1. Fruit intake ≥5 days/week  2. Vegetable intake ≥5 days/week  3. Physical activity ≥30 mins on ≥3 days/week  4. Never smoked tobacco  5. Blood sugar checked  6. Blood pressure checked  7. Discussed diet/Physical activity with healthcare workers. |
